# Breathing motion compensation in chest tomosynthesis: evaluation of the effect on image quality and presence of artifacts

**DOI:** 10.1117/1.JMI.12.S1.S13004

**Published:** 2024-09-14

**Authors:** Maral Mirzai, Jenny Nilsson, Patrik Sund, Rauni Rossi Norrlund, Micael Oliveira Diniz, Bengt Gottfridsson, Ida Häggström, Åse A Johnsson, Magnus Båth, Angelica Svalkvist

**Affiliations:** a Sahlgrenska Academy, University of Gothenburg, Department of Medical Radiation Sciences, Gothenburg, Sweden; b Sahlgrenska University Hospital, Department of Medical Physics and Biomedical Engineering, Gothenburg, Sweden; c Sahlgrenska Academy, University of Gothenburg, Department of Radiology, Gothenburg, Sweden; d Sahlgrenska University Hospital, Department of Radiology, Gothenburg, Sweden; e Chalmers University of Technology, Division of Signal processing and Biomedical Engineering, Department of Electrical Engineering, Gothenburg, Sweden

**Keywords:** chest tomosynthesis, motion artifacts, breathing motion, image quality, motion compensation

## Abstract

**Purpose:**

Chest tomosynthesis (CTS) has a relatively longer acquisition time compared with chest X-ray, which may increase the risk of motion artifacts in the reconstructed images. Motion artifacts induced by breathing motion adversely impact the image quality. This study aims to reduce these artifacts by excluding projection images identified with breathing motion prior to the reconstruction of section images and to assess if motion compensation improves overall image quality.

**Approach:**

In this study, 2969 CTS examinations were analyzed to identify examinations where breathing motion has occurred using a method based on localizing the diaphragm border in each of the projection images. A trajectory over diaphragm positions was estimated from a second-order polynomial curve fit, and projection images where the diaphragm border deviated from the trajectory were removed before reconstruction. The image quality between motion-compensated and uncompensated examinations was evaluated using the image quality criteria for anatomical structures and image artifacts in a visual grading characteristic (VGC) study. The resulting rating data were statistically analyzed using the software VGC analyzer.

**Results:**

A total of 58 examinations were included in this study with breathing motion occurring either at the beginning or end (n=17) or throughout the entire acquisition (n=41). In general, no significant difference in image quality or presence of motion artifacts was shown between the motion-compensated and uncompensated examinations. However, motion compensation significantly improved the image quality and reduced the motion artifacts in cases where motion occurred at the beginning or end. In examinations where motion occurred throughout the acquisition, motion compensation led to a significant increase in ripple artifacts and noise.

**Conclusions:**

Compensation for respiratory motion in CTS by excluding projection images may improve the image quality if the motion occurs mainly at the beginning or end of the examination. However, the disadvantages of excluding projections may outweigh the benefits of motion compensation.

## 1 Introduction

Chest tomosynthesis (CTS) is an X-ray examination that has been shown to be a promising imaging technique in chest radiology.^1^^–^^3^ Although conventional chest radiography (CXR) remains the most common diagnostic examination for pulmonary diseases, with the advantages of low cost and low radiation dose, CTS has shown an increased sensitivity in the detection of pulmonary nodules.^1^^,^^4^ The increased sensitivity in the detection of these lesions is due to the tomographic technique used in CTS resulting in section images of the chest where the impact of overlaying anatomy is reduced.^5^

A CTS examination is usually performed using the same system and the same patient setup as the ones used for CXR examinations. During a CTS examination, the patient is standing in front of the detector while the X-ray tube performs a continuous vertical movement, collecting several low-dose posteroanterior (PA) projection images of the chest, each with a slightly different angle.^3^^,^^4^^,^^6^^,^^7^ The projection images are then used to reconstruct section images of the chest using filtered back projection.^4^^,^^5^ The total examination time is longer for CTS compared with CXR, usually between 10 and 12 s.^8^^,^^9^ During this time, the patients are required to stand still and hold their breath to avoid motion artifacts in the reconstructed section images. In CTS, motion artifacts from the continuous and uncontrolled movement of thoracic organs, such as the heart, are relatively common and appear as blurring of nearby structures (vessels). While motion artifacts from, e.g., the heart appear in a limited region of the reconstructed section images, motion artifacts occurring motion artifacts occurring from patient breathing affect the entire image and thereby might impact the possibility of using the images for diagnostic purposes.^10^^,^^11^ Studies have shown that the presence of motion artifacts negatively affects the detection of pulmonary nodules.^10^^,^^12^^,^^13^ In one study focusing on nodule detection, it was shown that a relatively large lesion (17 mm in diameter) was not detected by any of the observers participating in the study.^12^

A CTS examination with severe motion artifacts present will most likely be non-diagnostic and might require the examination to be retaken, causing additional radiation dose to the patient. One study has addressed motion compensation in chest tomosynthesis based on the respiratory signal extracted from the location of the diaphragm in the projection images.^11^ The respiratory signal was used to divide the projection images into respiratory phases, and reconstruction of section images for each respiratory phase was made. This approach showed that the blur caused by motion artifacts could be reduced and was shown to be robust for respiratory signals with somewhat regular respiratory cycles but not robust enough for irregular respiratory signals.^11^ However, as patients undergoing CTS examinations are instructed to hold their breath during image acquisition, the accidental breathing motions are more likely to be irregular. The aims of the present study are therefore to (1) assess if motion artifacts can be reduced by removing projection images identified with breathing motion before the reconstruction of the section images and (2) evaluate if the general image quality is improved by motion compensation using this method.

## 2 Material and Methods

The Swedish Ethical Review Authority approved this study (2021-03857).

### 2.1 Image Acquisition

Chest tomosynthesis examinations included in this study were retrospectively selected from 2969 CTS examinations including both the projection images and the corresponding reconstructed section images. The examinations were collected from subjects included in a prospective population study for cardiovascular and pulmonary diseases, the Swedish CArdioPulmonary bioImage Study (SCAPIS).^14^ The CTS examinations were performed using the GE Definium 8000 system with the VolumeRAD option (GE Healthcare, Chicago, Illinois, United States). For all examinations, 60 low-dose projection images were collected in a vertical linear sweep of the X-ray tube in the caudocranial direction, using a sweep angle of 30 deg, a stationary detector, and an acquisition time of ∼11  s. A tube voltage of 120 kV and additional filtration of 3 mm Al + 0.1 mm Cu had been used. The tube load (mAs) for each projection image in CTS was constant and determined from the resulting mAs from a PA scout image, taken with automatic exposure control, multiplied by a factor of 10 (dose ratio 1:10), and evenly distributed among the 60 projection images.

### 2.2 Motion Analysis

**Fig. 1:**
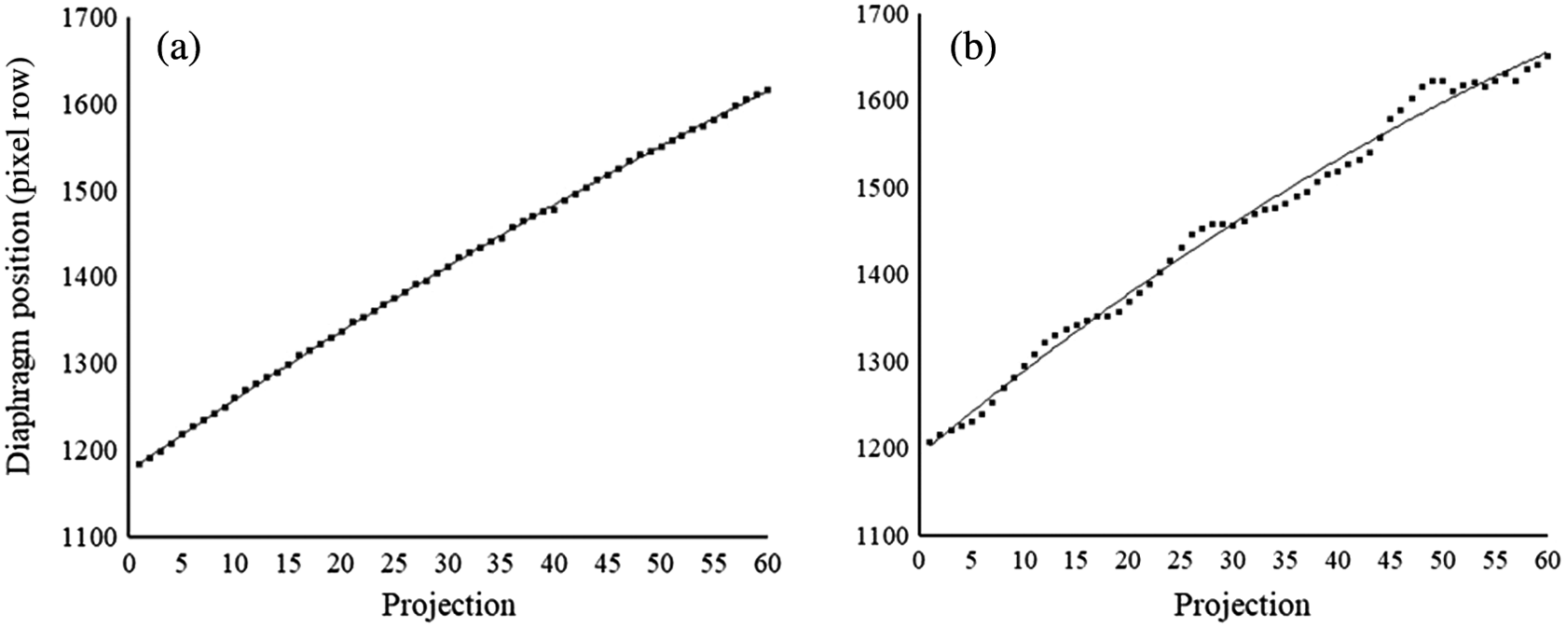
Examples of two CTS examinations where a second-order polynomial curve has been fitted to the position of the diaphragm border in each projection image (1 to 60). For a patient who stands still and holds their breath during the acquisition, (a) the position of the diaphragm shows almost no deviation from the trajectory, whereas for a patient with respiratory motion, (b) several deviations are seen.

As the X-ray tube performs a vertical linear movement during the collection of the tomosynthesis projection images, the location of a single point in the patient is depicted at different locations in each projection image. The displacement of the point among adjacent projection images should theoretically follow a vertical linear trajectory. However, as patient anatomy has an irregular shape and extends in three dimensions, the outline of the anatomy is not necessarily depicted with a linear displacement among the projection images. Therefore, a second-order polynomial function has previously been used to describe the moving trajectory of the diaphragm in CTS.^11^ To identify examinations where respiratory movement has occurred during the collection of the projection images, a method based on detecting the location of the diaphragm border in each of the projection images was used. In the method, a vertical movement of the diaphragm border was used as an indication of patient breathing. For example, for patients who are standing still and holding their breath during the examination, the displacement of the diaphragm border among the projection images normally follows a smooth non-linear trajectory. If the patient is breathing, or performing other irregular movements during image collection, a deviation from this smooth trajectory is found (Fig. 1).

### 2.3 Localization of Diaphragm

**Fig. 2:**
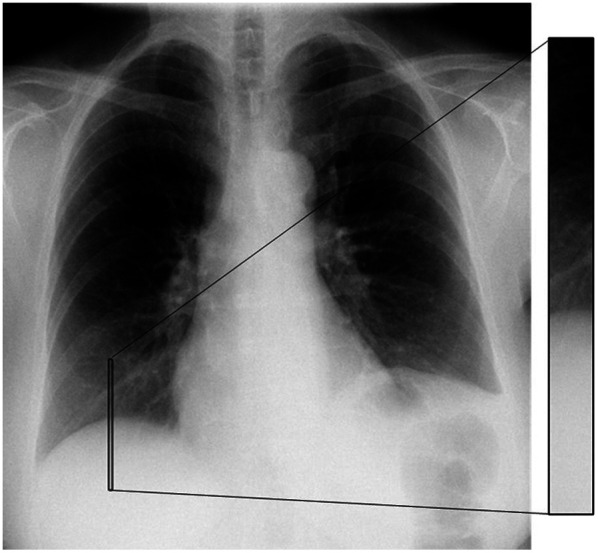
Example of the positioning of a fixed ROI over the diaphragm in one of the 60 projection images included in a CTS examination. The border of the diaphragm is detected using gradient analysis in the ROI.

To localize the diaphragm border, a region of interest (ROI) was placed at a fixed location in the right lung in each of the 60 projection images included in the CTS examination. In the ROI, the mean pixel value of each row was calculated to obtain a single column. The gradient within the column was calculated from top to bottom, and the location of the diaphragm border was estimated to be located at the vertical pixel position with the highest negative gradient. The vertical size of the ROI was 1000 pixels, which was estimated to be large enough to include the diaphragm border in all the projection images. The horizontal size of the ROI was set to 100 pixels to obtain descent statistics without introducing uncertainties due to the shape of the diaphragm border in the gradient analysis (Fig. 2). As the anatomy of the patients varies (e.g., due to different shapes of the diaphragm dome and patient size), the position of the ROI was manually reviewed to verify that the position was correctly placed over the border of the diaphragm. In cases where an erroneous position of the ROI was identified, the position was manually adjusted to ensure a correct analysis of the position of the diaphragm border in each projection image.

A trajectory of the position of the diaphragm border in each of the projection images included in an examination was obtained, and as no linear relationship exists among the displacement of the positions, a second-order polynomial curve was fitted to the positions.^11^ A deviation of the position of the diaphragm border from the obtained curve indicated motion. For a deviation of 10 pixels or more (corresponding to 2 mm or more with a pixel size of 0.2 mm), the motion was estimated to be considerable, and the corresponding projection image was removed before the reconstruction of the section images. This threshold was chosen based on the results from a study on CTS, where it was shown that the majority of patients had vertical or horizontal shifts of less than 2 mm during the acquisition of the projection images.^5^ After the removal of the projection images identified with motion, section images were reconstructed with a 5-mm slice interval using the reconstruction algorithm provided by the VolumeRAD system.

### 2.4 Inclusion Criteria

The inclusion criteria for the study were selected based on two primary considerations: (1) selecting examinations where breathing motion is present and where an improvement in image quality could be anticipated through motion compensation and (2) limiting the introduction of other image artifacts, mainly ripple and noise. Therefore, examinations were only included in the study if the number of identified projections to be removed was ≥5 or ≤35. The lower limit was chosen based on the assumption that the effect of compensating for breathing motion in such few projections was considered to have a minor impact on the resulting image quality in the reconstructed section images. In a previous study by Söderman et al.,^15^ it was shown that removing projection images from a CTS examination before reconstruction of the section images will increase the number of artifacts present in the reconstructed images. In the study, the comparisons of the different configurations of projection images (different projection densities and different angular intervals used for the collection of the projection images) were made using the same total effective dose to the patient. In the present study, the removal of projection images will, in addition to increased amounts of artifacts, also result in increased image noise. There have been studies showing that CTS, for some clinical indications, can be performed using lower dose levels (effective dose ∼0.05 to 0.06 mSv) without a significant reduction in diagnostic information due to the increased noise level in the images.^9^^,^^16^^–^^18^ The total radiation dose from a CTS examination is proportional to the number of projection images included in the examination. It has been shown that the effective doses for average-sized patients are ∼0.12  mSv for CTS and 0.05 mSv for CXR (including a PA image and a lateral image).^19^ As the original CTS examinations include the acquisition of 60 projection images, a reduction of the number of projection images to 35 will result in a CTS examination with an effective dose corresponding to 0.05 mSv. Therefore, the upper limit for study inclusion was set to the removal of a maximum of 35 projections.

### 2.5 Image Quality Evaluation

In the present study, examinations with projection images removed due to identified breathing motion before reconstruction of the resulting section images are referred to as compensated examinations. The same examinations but without any removal of projection images with identified motion are referred to as reference examinations. The evaluations were performed both on the complete study data and subgroups. Four different subgroups were defined according to both the number and the positions of removed projection images. Differences in image quality between compensated examinations and reference examinations were evaluated using visual grading characteristic (VGC) analysis.^20^

In the VGC analysis, the image quality was evaluated by four thoracic radiologists, three with more than 15 years of clinical experience in chest tomosynthesis. The image quality criteria used in this study included both the reproduction of anatomical structures (criteria 1 to 5) and the presence of image artifacts (criteria 6 to 8) (see Table 1). During the evaluation, large-sized vessels were represented by pulmonary and segmental arteries and veins, medium-sized vessels were represented by segmental to subsegmental arteries and veins located up to 2 cm from the pleura, and small-sized vessels were represented by vessels located within 2 cm from the costopleural border. For small-sized vessels inferior to the highest point of the diaphragmatic dome, the region for evaluation was limited to the right hemidiaphragmatic dome to exclude possible motion artifacts induced by heart motion. The visibility of anatomical structures was evaluated using a 5-grade scale going from confident that the criterion is fulfilled to confident that the criterion is not fulfilled. In the evaluation of image artifacts, the observers rated the presence and disturbance of different artifacts (motion artifacts—not induced by heart motion, ripple artifacts, and noise) using a 3-grade scale (absent, present but not disturbing, or present and disturbing). The VGC study was conducted using ViewDEX, a software tool designed specially to facilitate observer performance studies.^21^

**Table 1:** Quality criteria used for the evaluation of image quality and image artifacts. Large-sized vessels consisted of pulmonary and segmental arteries and veins, medium-sized vessels consisted of segmental to subsegmental arteries and veins up to 2 cm from the pleura, and small-sized vessels consisted of vessels located within 2 cm from the costopleural border.

	Image quality criteria
1.	Clear reproduction of the trachea, carina, and main bronchi
2.	Clear reproduction of the large- and medium-sized vessels
3.	Possibility to follow medium- to small-sized vessels through the volume
4.	Clear reproduction of the small-sized vessels as seen within 2 cm from the costopleural border in the parenchyma superior to the highest point of the diaphragmatic dome
	4a. Anteriorly
	4b. Laterally
	4c. Posteriorly
5.	Clear reproduction of the small-sized vessels as seen within 2 cm from the costopleural border in the parenchyma inferior to the highest point of the right hemidiaphragmatic dome
	5a. Posteriorly
	5b. Laterally
6.	Presence of motion artifacts (not induced by heart motion)
7.	Presence of ripple artifacts
8.	Presence of image noise

VGC is a rank-invariant and non-parametric method that allows statistical analysis to be performed on ordinal data.^20^ In this study, the software VGC Analyzer^22^ was used for statistical evaluation of the data from this multi-reader and multi-case VGC study. Using VGC Analyzer, the rating data for each image quality criterion from the compensated examinations were compared with the corresponding rating data from the reference examinations, and a VGC curve was generated. The area under the VGC curve (${\mathrm{AUC}}_{\mathrm{VGC}}$) was used as a measure for comparison of the observed image quality in motion-compensated examinations with the reference examinations. For multiple observers, the VGC Analyzer provides the averaged ${\mathrm{AUC}}_{\mathrm{VGC}}$ over the observers and provides the asymmetric 95% confidence interval using a bootstrapping (resampling) technique. In this study, the ${\mathrm{AUC}}_{\mathrm{VGC}}$ was obtained using the trapezoidal rule for curve fitting, and the statistical analysis was based on the fixed-reader situation. For image quality criteria 1 to 5 (reproduction of anatomical structures), an ${\mathrm{AUC}}_{\mathrm{VGC}}>0.5$ indicated higher ratings for the compensated examinations whereas an ${\mathrm{AUC}}_{\mathrm{VGC}}<0.5$ indicated higher ratings for the reference examinations. An ${\mathrm{AUC}}_{\mathrm{VGC}}=0.5$ indicated no difference in the ratings among the two groups. For the quality criteria regarding image artifacts and noise (criteria 6 to 8), an ${\mathrm{AUC}}_{\mathrm{VGC}}>0.5$ indicated that image artifacts were rated as less present and disturbing in the compensated examinations, and ${\mathrm{AUC}}_{\mathrm{VGC}}<0.5$ indicated that image artifacts were rated as more present and disturbing in the compensated examinations. An ${\mathrm{AUC}}_{\mathrm{VGC}}=0.5$ indicated no difference between the two groups. No statistically significant difference in the ratings between the compensated and uncompensated examinations was considered if the 95% confidence interval included the value 0.5.

## 3 Results

### 3.1 Motion Detection

**Fig. 3:**
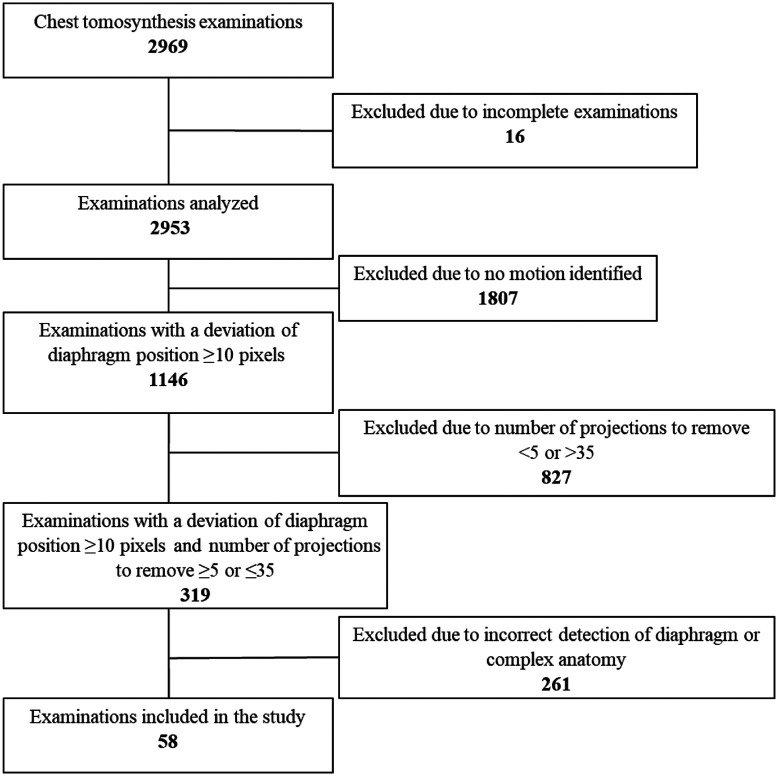
Flowchart describing the inclusion of examinations in the study.

The inclusion of examinations where motion was identified using the proposed method is shown in Fig. 3. Sixteen of the 2969 clinical CTS examinations were excluded prior to the motion analysis due to an incomplete number of projection images included in the examinations. From the remaining 2953 examinations, a total of 1146 examinations had at least one projection image where the position of the diaphragm deviated from the smooth trajectory, indicating breathing motion. From these, 827 were excluded as the number of projection images to be removed was <5 (n=819) or >35 (n=8). After a manual review and correction of the position of each ROI, it was shown that 261 of the 319 remaining examinations were falsely identified to include breathing motion due to erroneous detection of the diaphragm border due to overlapping anatomy. Finally, a total of 58 examinations were included in the study. Demographics for the patients included in the study are shown in Table 2.

**Table 2:** Patient demographics.

	**All examinations** (n=2953)	**Included in the study** (n=58)
**Gender**	N (%)	N (%)
Male	1529 (52)	32 (55)
Female	1424 (48)	26 (45)
**Demographics**	**Mean (std)**	**Mean (std)**
Age (years)	58 (4.3)	59 (3.9)
Weight (kg)	80 (15.3)	83 (15.4)
Height (cm)	172 (9.7)	172 (10.1)
BMI ($\mathrm{kg}/m^{2}$)	27 (4.3)	28 (5.3)
**Radiation dose**	**Mean (std)**	**Mean (std)**
Total DAP^a^ (${\mathrm{Gycm}}^{2}$)	0.58 (0.16)	0.63 (0.19)
Effective dose^b^ (mSv)	0.15 (0.04)	0.16 (0.05)

a Total dose area product (DAP) was extracted from the DICOM header of the projection images.

b The effective dose was determined using a conversion factor^19^ of $0.26\;\mathrm{mSv}\;{\mathrm{Gy}}^{-1}\;{\mathrm{cm}}^{-2}$.

**Fig. 4:**
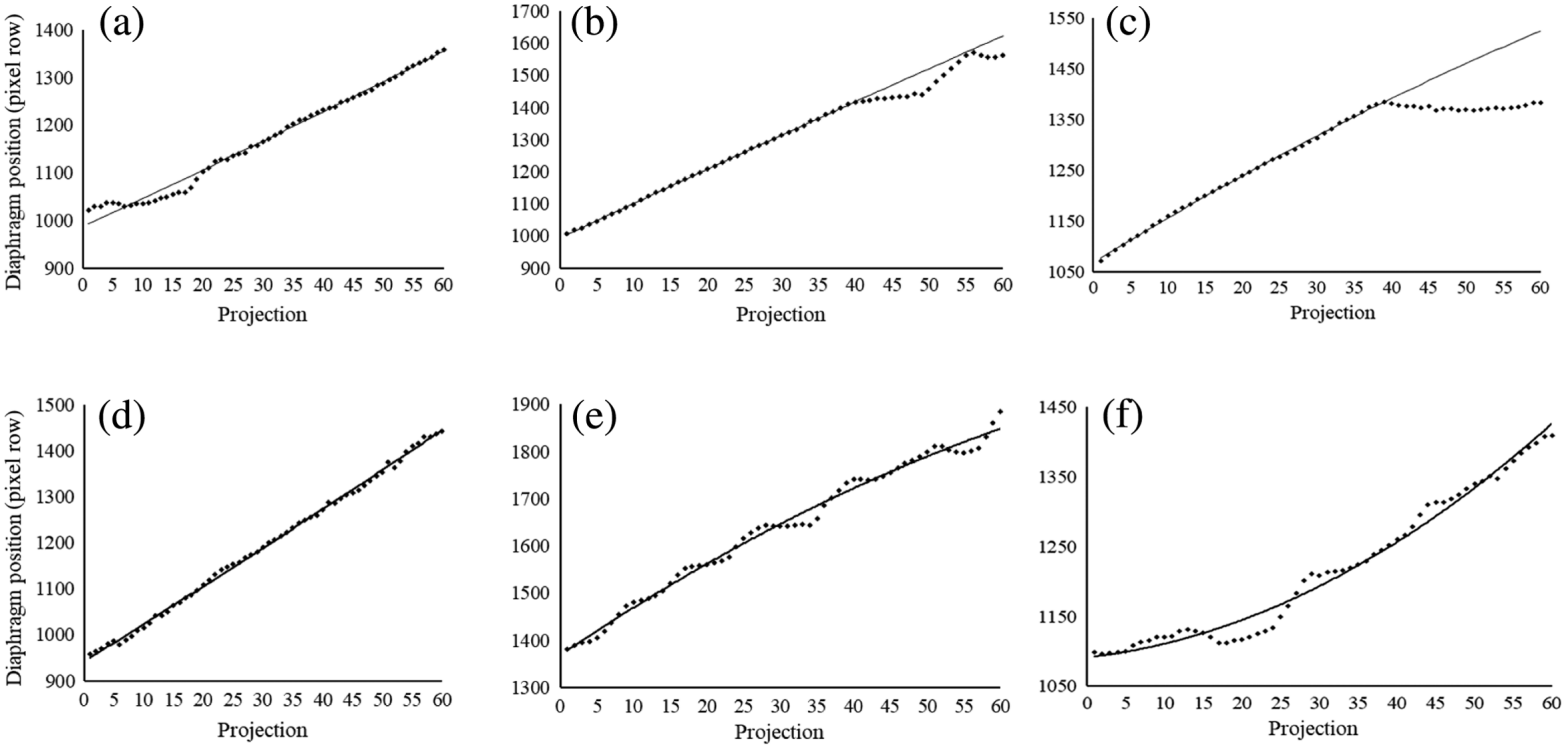
Examples of the diaphragm border positions with corresponding trajectories for six different examinations included in the study. In the first row [(a)–(c)], motion has been identified in either the beginning or the end of the trajectory. In the second row [(d)–(f)], motion is distributed over the entire trajectory.

The distribution of the number and position of the projection images that were removed due to identified breathing motion varied among the examinations included in this study. For some examinations, a motion was seen only in the beginning or in the end of the trajectory. However, for most of the examinations, projections with identified motion were more evenly distributed over the trajectory. In Fig. 4, the trajectories from examinations with different distributions of projection images with identified motion are presented. In the first row [(a) to (c)], examples of trajectories where motion was identified in either the beginning or the end of the examination are shown. The second row [(d)–(f)], shows examples of trajectories where projections with identified motion were distributed over the entire trajectory.

For a more detailed analysis based on the different distributions of projection images to be removed, the examinations were divided into four subgroups. Examinations with identified motion only at the beginning or at the end of the trajectory were appointed as subgroup A (n=17). For the rest of the examinations, the distributions of removed projections were more evenly distributed over the trajectory. These were further divided into three subgroups (B to D) based on the number of projection images that were removed due to motion (Table 3). Subgroup B included examinations where 5 to 11 projections were removed (n=17), subgroup C included examinations where 12 to 20 projections were removed (n=12), and subgroup D included examinations where 21 to 35 projections were removed (n=12).

**Table 3:** The classification of subgroups based on the distribution of the projection images identified with breathing motion. Subgroup A includes examinations where projections are removed only at the beginning or at the end of the trajectory, whereas subgroups B to D include examinations where the removed projections are evenly distributed over the trajectory (the difference among these groups is the number of projections removed). The range for the number of projection images removed is presented together with the total number of examinations in each subgroup.

Subgroup	n projections removed	n examinations
A	5 to 22	17
B	5 to 11	17
C	12 to 20	12
D	21 to 35	12
Total		58

### 3.2 Overall Image Quality Evaluation

**Fig. 5:**
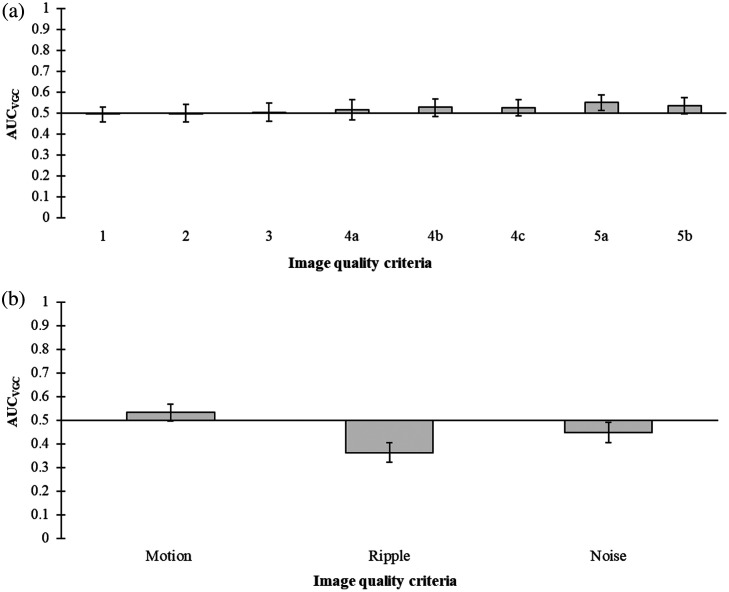
Resulting ${\mathrm{AUC}}_{\mathrm{VGC}}$ with the corresponding 95% confidence interval for all examinations included in the study (n=58). In (a), results for the anatomical quality criteria are presented, and ${\mathrm{AUC}}_{\mathrm{VGC}}>0.5$ indicates higher ratings in the compensated examinations, whereas ${\mathrm{AUC}}_{\mathrm{VGC}}<0.5$ indicates higher ratings in the reference examinations. The quality criteria related to image artifacts are shown in (b), where ${\mathrm{AUC}}_{\mathrm{VGC}}>0.5$ indicates less present and disturbing artifacts in the compensated examinations, whereas ${\mathrm{AUC}}_{\mathrm{VGC}}<0.5$ indicates more present and disturbing artifacts in the reference examinations.

The VGC analysis of the complete study data (n=58) showed, in general, no significant differences in the reproduction of anatomical structures between the compensated examinations and the reference examinations [Fig. 5(a)]. The only exception was found for the small-sized vessels located posteriorly in the region inferior to the highest point of the right hemidiaphragmatic dome (criterion 5a), which was rated significantly higher in the compensated examinations. Regarding the image artifacts, no significant difference was found between the compensated examinations and the reference (non-motion compensated) examinations, whereas ripple artifacts and noise were rated as significantly more disturbing in the motion-compensated examinations than in the reference examinations [Fig. 5(b)].

### 3.3 Subanalysis of Image Quality

**Fig. 6:**
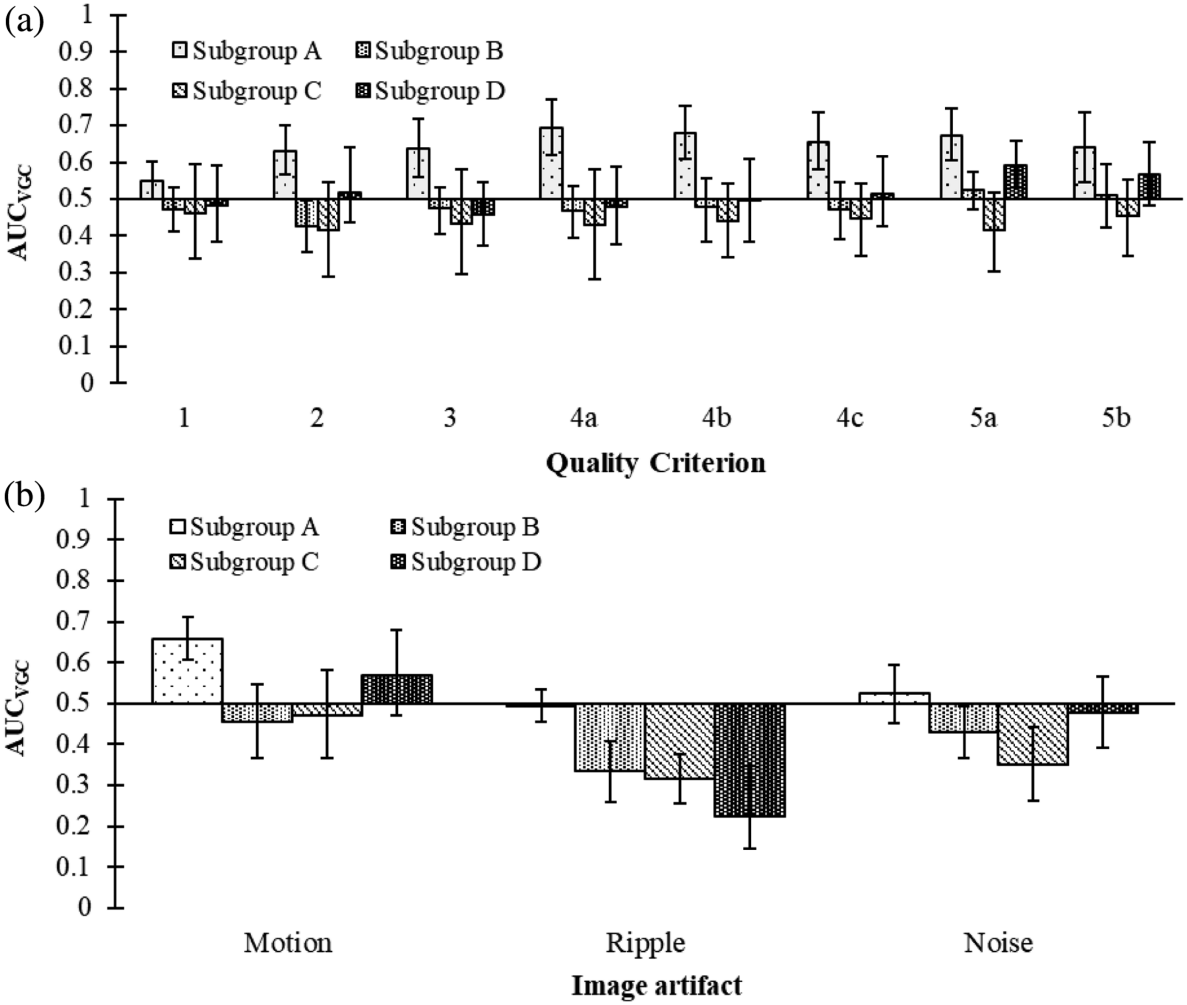
Resulting ${\mathrm{AUC}}_{\mathrm{VGC}}$ with corresponding 95% confidence interval for subgroups (A to D). In (a), results for the anatomical quality criteria are presented, and ${\mathrm{AUC}}_{\mathrm{VGC}}>0.5$ indicates higher ratings in the compensated examinations, whereas ${\mathrm{AUC}}_{\mathrm{VGC}}<0.5$ indicates higher ratings in the reference examinations. The quality criteria related to image artifacts are shown in (b), where ${\mathrm{AUC}}_{\mathrm{VGC}}>0.5$ indicates less present and disturbing artifacts in the compensated examinations, whereas ${\mathrm{AUC}}_{\mathrm{VGC}}<0.5$ indicates more present and disturbing artifacts in the reference examinations.

For examinations where projection images were removed in the beginning or in the end of the trajectory (subgroup A), the compensated examinations obtained significantly higher ratings for all image quality criteria [Fig. 6(a)]. For examinations where the removed projection images were distributed over the motion trajectory (subgroups B to D), no significant differences in the image quality criteria ratings were found, except for criterion 5a in subgroup D. In this subgroup, small-sized vessels located posteriorly in the region inferior to the highest point of the right hemidiaphragmatic dome were rated significantly higher in compensated examinations than in the reference examinations.

In subgroup A, motion artifacts were rated to be significantly less present and disturbing in the compensated examinations than in the reference examinations, whereas no significant differences were found in subgroups B to D. A significant increase in the presence and disturbance of ripple artifacts was found for compensated examinations in subgroups B to D, whereas no difference between compensated and reference examinations was noted in subgroup A. In addition, a significant increase in the presence and disturbance of noise was found for compensated examinations in subgroups B and C, whereas no differences were found in subgroups A and D.

## 4 Discussion

### 4.1 Motion Detection

CTS examinations identified with breathing motion were included in the study with the purpose of investigating if compensation of the breathing motion had a positive effect on the resulting image quality. To compensate for breathing motion, projection images identified with motion were removed prior to reconstruction of the section images. The image quality in compensated and uncompensated examinations was evaluated in a VGC study. Overall, no significant difference in image quality was observed between compensated and uncompensated examinations. However, in cases where the breathing motion occurred in the beginning or the end of the examination, compensation of breathing motion might be beneficial for the resulting image quality.

The method used for identifying patient breathing motion, together with the inclusion criteria used in the study, resulted in the inclusion of only two percent (58 out of 2953) of the available CTS examinations. The relatively low proportion of examinations identified with breathing motion can be explained by the fact that the examinations were from study subjects participating in a population study (SCAPIS), including randomly selected men and women aged 50 to 64 years.^14^ The study population can therefore be assumed to include individuals who, on average, are healthier than the patients who clinically undergo CTS examinations. Consequently, the study subjects can be expected to have a better ability to hold their breath and stand still during image acquisition compared with, for example, patients with known pulmonary disease.

The large proportion of examinations falsely identified to include motion using the method described in the present study (261 of the 319 examinations) was mainly due to an erroneous detection of the diaphragm border. The reason for the erroneous detection was that, in the method, the ROI was placed at a fixed position for all examinations in the initial automatic analysis. This approach did not consider the anatomical variations of the shape of the diaphragm or the presence of overlapping anatomy among different study subjects. For example, for some study subjects, the largest negative gradient in the ROI was sometimes found to be at the position of an overlapping vessel or rib instead of the diaphragm border. After manually adjusting the lateral position of the ROI for these study subjects, a better detection of the diaphragm border was achieved, and the examinations that thereafter fulfilled the inclusion criteria were included in the study.

In the proposed method, a trajectory of the diaphragm border positions among the projection images was obtained by fitting a second-order polynomial curve to the positions. The trajectory was used to estimate the expected diaphragm position in each projection image in the absence of respiratory motion. A deviation of the diaphragm position from the obtained trajectory was used as an indication of motion. In examinations with considerable breathing motion in the beginning or end of the examination (indicated by a large deviation of the diaphragm position in the beginning or end of the trajectory), the curve fit was affected by the deviating values. Therefore, in the cases where considerable motion was present in the beginning or the end of the trajectory, only the positions that were not clearly deviating from the smooth trajectory were accounted for in the curve fitting.

### 4.2 Overall Image Quality Evaluation

The overall image quality evaluation for all examinations (n=58) showed no significant differences in ratings between compensated and reference examinations, except for criterion 5a (Fig. 5). Regarding the ratings of image artifacts, no significant difference was found for the presence and disturbance of motion artifacts between the compensated examinations and the reference examinations. Ripple artifacts and noise were however rated significantly more disturbing in the compensated images than in the reference images. These results can be expected, as removing projection images before reconstruction of the CTS section images results in (1) a reduced projection density, causing ripple artifacts, and (2) reduced total radiation dose, causing increased amounts of quantum noise.^15^^,^^23^^,^^24^ One reason why motion compensation did not improve neither the presence of motion artifacts nor the reproduction of anatomical structures could be that the significant increase in ripple artifacts and noise obscured the effects of motion compensation. The reproduction of small-sized vessels located posteriorly in the region inferior to the highest point of the right hemidiaphragmatic dome (criterion 5a) did show an improvement with compensation. One reason for this result could be that the displacement of anatomy due to breathing is more prominent in the area close to the diaphragm border,^25^ why the benefit of compensation on image quality is larger for structures located in this area.

### 4.3 Subanalysis of Image Quality

In the subanalysis, the effect of breathing motion compensation on image quality was analyzed in subgroups based on the distribution and number of removed projection images. In subgroup A (Fig. 6), where the removal of projection images only occurred in the beginning or in the end of the diaphragm position trajectory, a significant improvement in image quality was found in the compensated examinations compared with the reference examinations. Removing the projection images in the beginning or in the end of the trajectory should not lead to an increase in ripple artifacts as the projection density is unaffected. Instead, this situation corresponds to a decreased angular range for the collection of the projection images, which instead affects the depth resolution in the reconstructed section images.^15^^,^^23^^,^^24^ Theoretically, the possibility to follow medium- to small-sized vessels through the volume (criterion 3) should have been rated lower in the compensated examinations as this criterion can be used to assess the depth resolution in the examinations. However, this criterion was rated higher in the compensated examinations than in reference examinations. This might be due to the fact that the blurring of the vessels caused by breathing motion negatively affected the possibility of following the vessels throughout the volume, which could explain why this criterion was rated higher in the compensated examinations. In this subgroup, the presence of motion artifacts was rated as significantly less present and disturbing in the compensated examinations. The ratings regarding the presence and disturbance of ripple artifacts and noise showed no significant difference between the compensated examinations and the reference examinations, indicating that the presence of motion artifacts could be compensated for without impairing the image quality [Fig. 6(b)]. The fact that no difference was found regarding the presence of ripple artifacts was expected as the projection density in subgroup A was unaffected by the removal of projection images. Regarding noise, it was expected that the presence of noise would be rated higher in the compensated examinations due to the removal of projection images.^23^ However, no significant difference in the ratings of noise was found between the compensated examinations and the reference examinations in this subgroup. Possibly, this is related to that the decreased angular range in the compensated examinations also results in a decreased depth resolution.

The evaluation of the image quality in the other subgroups (B to D), where the removed projection images were distributed across the trajectory, showed no significant difference in ratings between the compensated examinations and the reference examinations, except for criterion 5a in subgroup D [Fig. 6(a)]. A significant increase in ripple artifacts was observed in all these subgroups (B to D). This finding corresponds to the results in previous studies, where it has been shown that a lower projection density leads to an increase in ripple artifacts.^15^^,^^23^^,^^24^ In the present study, the increase in ripple artifacts might affect the image quality in such a way that compensation of breathing motion using the described method might not be advantageous. A significant increase in noise was found for subgroups B and C but not in subgroup D. This was unexpected as a larger proportion of projection images were removed in subgroup D than in subgroups B and C. However, as the projection density is more reduced in this subgroup compared with the other subgroups, larger amounts of ripple artifacts are introduced in this subgroup [Fig. 6(b)]. This could lead to a situation where the presence of noise in the images is obscured by the ripple. The fact that ripple artifacts are more prominent in the compensated examinations for subgroups B to D compared with subgroup A [Fig. 6(b)] might explain why no significant differences are found in ratings of the anatomical quality criteria in subgroups B to D [Fig. 6(a)].

### 4.4 Limitations

The primary aim of the study was not to develop a robust method for identifying examinations with breathing motion, and the method used in the study therefore was not optimized for the task. It was merely used as a tool to automatically go through a relatively large number of examinations and exclude examinations where no breathing motion was present to limit the number of examinations that needed to be reviewed manually. When analyzing breathing motion in the present study, the position of the diaphragm border was considered the main anatomical marker for respiration. A limitation of this method is that, given the variations in the shape of the diaphragm among individuals, it can be expected that the position on the diaphragm border used for motion analysis must be manually adjusted among different individuals. However, manual adjustments were also needed to ensure correct detection of the diaphragm border and correct curve fitting of the trajectories. Without manual adjustments, the method would include a relatively large number of examinations falsely identified with motion. Another limitation of the method is that it may not identify slow relaxation of the diaphragm or poor quality “leaky” breath holds, as the smooth motion of the diaphragm may be mistaken for anatomical curvature by the method. Therefore, this type of continuous motion might potentially be present in the study population without being detected by the method. This might also explain the relatively low rate of patients with identified motion in the study (two percent). In addition, as the smooth motion of the diaphragm still results in a smooth trajectory that the curve fit will adapt to, fewer projections than those actually affected by motion will deviate from the curve fit, possibly resulting in an under-compensation of motion in these examinations. This might be one reason why no overall significant difference in image quality was found between compensated and reference examinations in the study. One way to overcome this problem could be to compare the motion of the diaphragm border to the shift in the position of another anatomical structure present in the images. Preferably, the anatomical structure chosen for such a comparison should be a structure not heavily affected by patient breathing.

Assessment of image quality was based on the fulfillment of the image quality criteria. It could be argued that there would be an additional clinical value in evaluating the effect on the detection of pathology rather than on the visibility of anatomical structures. However, if the quality criteria used include clinically relevant structures, visual grading studies have high validity.^20^ The image quality criteria used in this study have been established by a group of thoracic radiologists with long experience in clinical CTS. The criteria have been adapted and modified to CTS from the European guidelines on the image quality criteria for CXR and CT.

Another limitation of the study is the relatively small number of examinations included in each subgroup (Table 3). For all subgroups, the number of examinations was less than 20, which decreases the possibilities to detect significant differences between compensated and uncompensated examinations as the confidence interval becomes larger due to the low number of cases. However, despite this limitation, a significant difference in image quality was found for subgroup A.

In tomosynthesis, projection images are collected within a relatively small angular range, resulting in a limited sampling of the frequency domain. As a consequence, image reconstruction in tomosynthesis may be challenging. Many different methods have been developed with the purpose of reducing the problem with artifacts resulting from the limited sampling of the frequency domain.^26^ In the present study, all image reconstruction was performed using the reconstruction algorithm provided by the VolumeRAD system, which is based on filtered backprojection.^27^ This reconstruction algorithm is adapted to the clinically used acquisition parameters. When altering the angular range and projection density, as in the present study, the use of this default reconstruction algorithm might be suboptimal. If a reconstruction algorithm adapted to the new conditions would be used instead, the reconstructed images might include fewer artifacts, which could have an impact on the result from the present study.

## 5 Conclusion

Compensating for motion artifacts in CTS by removing projection images with identified breathing motion before reconstruction might be beneficial if the motion occurs only in the beginning or the end of the examination. If breathing motion is present throughout the examination, the method introduces ripple artifacts that possibly obscure the positive effects of motion compensation.

## Data Availability

The data utilized in this study were collected from the subjects included in a prospective population study, the SCAPIS, and are available from the authors upon request, with permission from SCAPIS. The code related to the method used in this study is available upon request from the corresponding author.
